# Supplementary material for: Inflammatory factors and risk of lung adenocarcinoma: a Mendelian randomization study mediated by blood metabolites
Source: Front Endocrinol (Lausanne). 2024 Aug 27;15:1446863. doi: 10.3389/fendo.2024.1446863 (PMC11384989; doi:10.3389/fendo.2024.1446863)
Supplement: Supplementary file 1 [file DataSheet1.zip › supplementary files/S2/GCST90200134/forest.pdf]

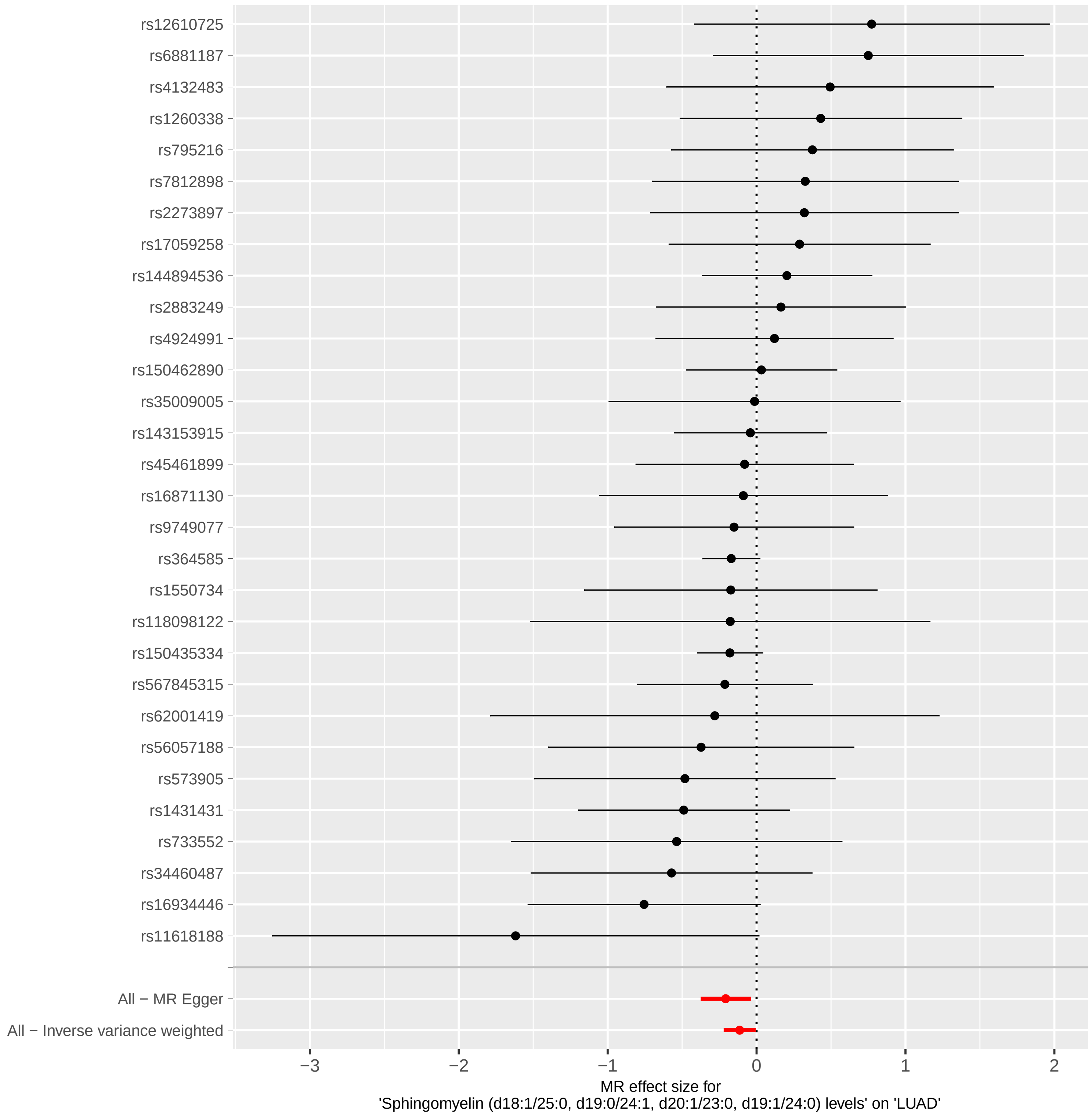

rs12610725
rs6881187
rs4132483
rs1260338
rs795216
rs7812898
rs2273897
rs17059258
rs144894536
rs2883249
rs4924991
rs150462890
rs35009005
rs143153915
rs45461899
rs16871130
rs9749077
rs364585
rs1550734
rs118098122
rs150435334
rs567845315
rs62001419
rs56057188
rs573905
rs1431431
rs733552
rs34460487
rs16934446
rs11618188
All − MR Egger
All − Inverse variance weighted
−3
−2
−1
0
1
2
MR effect size for
'Sphingomyelin (d18:1/25:0, d19:0/24:1, d20:1/23:0, d19:1/24:0) levels' on 'LUAD'
